# Review of the OSHA-NIOSH Response to the Deepwater Horizon Oil Spill: Protecting the Health and Safety of Cleanup Workers

**DOI:** 10.1371/4fa83b7576b6e

**Published:** 2012-07-18

**Authors:** David Michaels, John Howard

## Abstract

Introduction: The fire and explosion of the Deepwater Horizon oil rig resulted in an enormous oil spill that threatened large distances of coastline. The overall response was led by the United States Coast Guard and involved the oil company BP, federal agencies, and state and local governments of five states. Methods: The Occupational Safety and Health Administration and the National Institute for Occupational Safety and Health focused extensive resources on ensuring that BP and its contractors provided safe working conditions for thousands of workers involved in the response. Federal personnel visited worksites daily, identifying hazards and means of abatement; assessed training programs to ensure that workers were adequately trained in languages they could understand; monitored chemical exposures and determined that the proper personal protective equipment was deployed; insisted on implementation of a heat mitigation program; rostered thousands of workers; and conducted extensive outreach in communities impacted by the spill. Results: Advance planning, immediate deployment, and collaboration across agencies helped ensure that the response operations resulted in no worker fatalities, and relatively few injuries and illnesses. Conclusions: For future responses, improvements should be made in how safety and health information, as well as the process behind safety and health decisions, are communicated to the public. Citation: Michaels D, Howard J. Review of the OSHA-NIOSH Response to the Deepwater Horizon Oil Spill: Protecting the Health and Safety of Cleanup Workers. PLoS Currents Disasters. 2012 Jul 18

## The Deepwater Horizon Spill

‘On the night of April 20, 2010, the Deepwater Horizon oil rig, located 72 kilometers offshore southeast of Venice, Louisiana, exploded and caught fire, resulting in the deaths of eleven workers. It soon became clear that the Macondo wellhead, 1,500 meters below the ocean surface, was discharging crude oil into the Gulf of Mexico. The rig, operated by the oil company BP, sank on the morning of April 22, 2010. Many millions of barrels of oil discharged into the Gulf before the leak was sealed on July 15, 2010, making it the largest oil spill in U.S. history and the first to be declared a Spill of National Significance.^1^

Faced with an environmental catastrophe that threatened hundreds of kilometers of coastline, BP hired tens of thousands of workers, many unconditioned to the heat and long hours of work and with limited experience responding to and cleaning up oil spills. At the height of the response, the workforce totaled more than 47,000 men and women, including over 42,000 response and cleanup workers employed by BP and its contractors, 1,600 members of the National Guard, and more than 2,400 federal employees. The government response was unprecedented in its scope and duration, involving many federal agencies and the state and local governments of five states: Louisiana, Mississippi, Alabama, Florida, and Texas.

## Overview of the response

Federal responses to oil spills are conducted under the National Oil and Hazardous Substances Pollution Contingency Plan (also known as the National Contingency Plan or NCP), which establishes a multi-tiered National Response System. Under the NCP, the National Response Team (NRT), a standing group of 16 federal agencies, is responsible for interagency preparedness and response for oil and hazardous materials releases. The NRT, which includes the Occupational Safety and Health Administration (OSHA) and the National Institute for Occupational Safety and Health (NIOSH), began meeting to plan the response to the oil spill on April 22, 2010, the same day the rig sank.

After the oil spill was declared a Spill of National Significance on April 29, a National Incident Command (NIC) was established to coordinate resources at the national level. The NIC was under the leadership of the United States Coast Guard (USCG) because the spill occurred in coastal waters. Local incident/unified command posts were set up to coordinate multiagency operations. These were supervised by the Unified Area Command, consisting of the On-Scene Coordinator (from the USCG) and the responsible party (in this case, BP).^2^ OSHA and NIOSH provided guidance and safety and health expertise to all levels of the Unified Command: the local incident command posts; the Unified Area Command; and the National Incident Command.

The Unified Command provided a structure for agencies from across the federal government to work together, fostering collaboration between a range of agencies with public health and worker protection responsibilities. These agencies included OSHA, NIOSH, USCG, the National Institute of Environmental Health Sciences (NIEHS), the Environmental Protection Agency (EPA), the National Oceanic and Atmospheric Administration (NOAA), the Department of Labor’s Employment and Training Administration and Wage and Hour Division, the Food and Drug Administration, and the Substance Abuse and Mental Health Services Administration.

Under the Occupational Safety and Health Act, BP was responsible as an employer for providing a safe and healthful workplace for response workers hired by BP and its contractors.^3^ BP was also obligated under the NCP to implement a safety and health program consistent with OSHA standards.^4^

OSHA began planning its operations in the Gulf within days of the explosion and invited NIOSH to join the interagency operational effort. As the Unified Command opened staging areas and BP hired workers to prepare for cleanup activities, OSHA assigned staff to ensure that BP adequately trained, equipped and protected the cleanup workers. OSHA recognized the need to communicate with workers in their native languages, and deployed staff fluent in Vietnamese and Spanish to aid the oil spill response.

Throughout the response, nearly 150 OSHA professionals were involved in protecting workers in the Gulf Region, with 25 to 40 of them assigned full-time to the oil response cleanup. OSHA personnel were active in all 17 staging areas in Louisiana, Mississippi, Alabama, and Florida. They visited worksites each day to assess whether BP was following OSHA guidance and providing appropriate worker safety and health protections. OSHA staff made over 4,200 site visits, covering: staging areas; decontamination, distribution, and deployment sites; and “Vessels of Opportunity,” the displaced fishing vessels involved in defensive booming, transporting work crews, and other types of support. When OSHA identified safety and health hazards, these concerns were communicated to the Unified Command. This approach helped ensure that hazards were identified and fixed and that consistent incident-wide protective measures were implemented.

Well before the oil reached the shore, OSHA assisted the Unified Command with conducting Job Hazard Analyses for the tasks it was anticipated workers would be performing and identified the protections required in a comprehensive safety and health program. OSHA also provided guidance for the development of a heat stress mitigation program and strongly encouraged BP to keep a comprehensive incident-wide log to record the full spectrum of worker injuries and illnesses. This included traditional OSHA recordable cases, which require treatment beyond first aid or lead to restricted duty or missed days of work, as well as “near misses” and injuries that only required first aid. BP reported these OSHA recordable and non-recordable injuries and illnesses during daily conference calls.

From May 3 through mid-August, NIOSH deployed over 100 staff to multiple locations in the five-state Gulf region and provided subject matter expertise to the Unified Area Command. NIOSH activities included rostering over 55,000 workers, conducting on- and off-shore health hazard evaluations, providing technical guidance and communication/educational materials, analyzing health surveillance information, and performing toxicity testing on samples of the oil dispersant and the crude from the leaking well. In total, close to 250 NIOSH staff participated in the response in some capacity.

**Assessment and Control of Chemical Exposures.** From the start of the response, the Unified Command was concerned about the potential health effects on workers from inhalation and skin exposure to crude oil, weathered oil, dispersants, solvents used to clean boats, and other chemicals. OSHA provided input to help BP develop a sampling strategy to address these hazards. OSHA also developed its own sampling protocol and strategy, based on a review of the specific tasks workers would be performing, the chemicals of concern from the oil and dispersants, and available Occupational Exposure Limits (OELs). The sampling strategy OSHA developed to support the Unified Command incorporated onshore zones, near-shore, and offshore operations.^5^

On May 6, OSHA deployed its specialized industrial hygienist team to Louisiana to provide technical support for the agency’s extensive air monitoring for potential worker chemical exposures at staging areas and on vessels.

A specialized committee convened each morning, consisting of the lead industrial hygienists from OSHA, NIOSH, USCG, EPA, the U.S. Department of the Interior, and BP and its contractors to review and discuss the results of air monitoring conducted by BP and the agencies. This group discussed anomalies from the previous day’s samples, reviewed updates to safe work practices and administrative controls, coordinated their sampling activities for the day, and identified safety and health concerns that needed to be addressed.

During the response, BP conducted more than 100,000 exposure assessments. OSHA reviewed these assessments and validated them by performing more than 7,000 independent worker exposure measurements, both onshore and on marine vessels. OSHA posted its data on the web,^6^ and strongly encouraged BP to do the same, providing the public with unprecedented access to exposure monitoring data.^7^

Outside of the source of the crude oil discharge, the exposure monitoring data showed that chemical exposure levels were mostly well below OELs. The source of the release was approximately 72 kilometers offshore. As the result of sun, water and wave action, and other environmental factors, most of the toxic volatile components of the oil dissipated during the weeks it took to reach the shoreline and other work areas of most cleanup workers. Based upon the monitoring data, OSHA and NIOSH recommended that respirators should be considered the protection of last resort, as they can be physically taxing on the body and therefore potentially dangerous, particularly for workers who have not used them before and in conditions of extreme heat.

However, for certain operations, such as decontamination or activities near the source to control the oil spill and cap the well, the exposure monitoring data were more varied, and in some cases exceeded the most protective OELs. In anticipation of these hazards, OSHA and NIOSH had encouraged the Unified Command to issue guidance recommending the use of respiratory protection for workers involved in these operations.^8^ The evidence from the sampling data supported the conclusion that respiratory precautions were important for workers engaging in decontamination operations or working near the source of the oil spill. OSHA confirmed that the respiratory protection recommendations were implemented.

On May 28, 2010, BP requested that NIOSH conduct Health Hazard Evaluations (HHEs) for Deepwater Horizon responders involved in both on- and off-shore response activities. The objective of the HHEs was to collect independent exposure and health symptom information from response workers in six work task categories: (1) source control activities; (2) off- shore activities; (3) shoreline clean-up activities; (4) decontamination activities; (5) wildlife cleaning; and (6) waste stream management activities. As part of the HHEs, NIOSH coordinated and conducted the analysis of hundreds of worker breathing zone air samples and other industrial hygiene samples and obtained information on responder health symptoms and injuries. In addition to obvious potential chemical exposures, other potential exposures such as heat stress, ergonomic factors, carbon monoxide exposure, and psychological stress were also addressed. The findings and recommendations were reported and posted to the NIOSH website.^9^

**Protecting Workers from the Hazards of Heat.** Perhaps the most serious health hazard faced by response and cleanup workers was heat. Many people, some wearing chemical-resistant Tyvek® coveralls, boots, and gloves, worked 12 hours a day, seven days a week in the hot and humid weather. From the outset, OSHA insisted that BP implement a robust program to protect workers from the heat illnesses; the program included work/rest requirements, shaded rest areas, hydration liquids, and onsite heat monitoring. The application of work/rest requirements was one of the most controversial of approaches applied, as some observers, unfamiliar with the heat/rest matrix, questioned why workers were often seen resting in shaded areas.

The Unified Area Command utilized existing USCG and the American Conference of Governmental Industrial Hygienists (ACGIH) heat stress protocols to publish a Heat Stress Alert on June 8, 2010.^10^ This Alert provided workload/rest cycles and other recommendations for working in the heat. This Alert was followed-up with the Unified Area Command’s Heat Stress Management Plan which provided additional guidance for managing the effects of heat exposure.^11^

Even though on many days the temperature reached above 100° F (37.8° C) no workers involved in the clean-up and response developed serious heat illness. Heat did, however, pose a significant hazard: as of September 2, when OSHA began transitioning out of the spill response, there were 978 heat stress incidents reported. The heat protection program likely prevented serious heat illnesses and possible deaths among workers.

**Training.** Worker training was an immediate priority because of the large number of workers engaged in the response, and the wide variety of tasks these workers were performing. OSHA and NIEHS collaborated on training publications, materials, and oversight of training programs.^12^ Well before oil reached the shore, OSHA established requirements for training for all response workers, and made it clear to BP that all workers would need to receive free training provided in a manner and language that workers understood. This training included specific requirements for personal protective equipment (PPE) for the different job tasks.

As the training requirements were identified, BP created a series of courses, including a one-hour orientation, a four-hour shoreline course, and four and eight-hour marine cleanup courses. OSHA, NIOSH, NIEHS and USCG reviewed BP’s proposed training program. BP launched a multi-tiered training program on May 5.

By May 21, the day after the first tar balls washed ashore on coastal Louisiana,^13^ approximately 10,000 workers along the shoreline and in boats had been trained. Ultimately, in the course of the response, more than 100,000 workers along the Gulf Coast received training on how to work safely.

Worker training was provided in English, Spanish, and Vietnamese. OSHA continually monitored training sessions, and compliance officers conducted on-site visits at staging areas and work sites to confirm that workers received appropriate training in a format and language that the workers could understand.

At OSHA’s request, BP instituted a credentialing program, whereby workers received certificates after completing training. All workers engaged in response and cleanup activities had to possess a training certification card showing that they had completed the BP-sponsored training program. During site visits, OSHA personnel interviewed workers about the training they had received and verified that workers had training cards. This ensured that site control was maintained and that only individuals with the proper training were present at worksites.

**Rostering.** A lesson learned from the World Trade Center attack was the difficulty of retrospectively creating a listing of response workers. During the Deepwater Horizon response, NIOSH developed a prospective roster of workers and volunteers to: (1) record those who participated in response activities and the training received before engaging in response work; (2) serve as a repository of information on the nature of each responder’s work assignments during the response; and (3) facilitate contact with responders during and after the response about possible work-related symptoms of illness or injury, as needed.^14^

Many workers were rostered during official safety training prior to being hired. However, by the time the roster was initiated, many other workers had already been trained and sent to the field. This necessitated an intensive effort of deploying NIOSH staff across the Gulf Coast to worker staging areas in Louisiana, Mississippi, Alabama, and Florida where rostering could also be accomplished. Ultimately over 55,000 workers were successfully rostered.

**Public and Community Outreach.** The Department of Labor (DOL) made a concerted effort to reach out to the communities impacted by the spill to provide assistance. DOL’s Employment and Training Administration worked with the One-Stop Career Centers in the affected area to help facilitate the hiring and training of displaced workers. The Employment and Training Administration published guidance and regularly communicated with state and local agencies. OSHA included information on the One-Stop Career Centers in its communication materials and highlighted them during community meetings. OSHA ensured that these workers, many of whom had no prior experience or training for cleaning up oil spills, were made aware of the protective equipment and training they needed to safely perform response and cleanup operations.

Outreach staff, including Spanish and Vietnamese speaking OSHA personnel, attended town halls and smaller community meetings; special efforts were made to meet with organizations representing immigrant residents and workers. In addition, OSHA’s area offices and its national call center fielded hundreds of calls and answered workers’ questions or directed them to the proper resources. These outreach efforts identified issues and concerns, such as when there was a lack of training in the workers’ primary languages.

OSHA and NIOSH also devoted significant resources toward maintaining constant communication with elected representatives, community leaders and stakeholders. The agencies participated in daily intergovernmental conference calls to provide worker safety and health updates to governors in the affected states, mayors and local elected officials, and members of Congress, and hosted or joined in weekly conference calls with state health departments, environmental organizations, worker organizations, and academic experts. In addition, OSHA and NIOSH personnel were interviewed by print and electronic media throughout the response.

Overall, the efforts to ensure the safety and health of these cleanup workers were very effective. There were no work-related fatalities. NIOSH reported that between April 23 and July 27 there were 1136 injuries and 994 illnesses. Of these, 175 injuries and 106 illnesses were OSHA-recordable cases. Because protection efforts were so effective, few safety and health issues emerged as significant concerns in the media at the national level. Although there is no evidence of significant short term health effects, there is a possibility of long term effects, even with the low exposure levels. This is the subject of a study being coordinated by NIEHS.^15^ There were both successes and challenges in the Deepwater Horizon response and the corresponding knowledge gained should be institutionalized for future emergency responses.

## Observations

**Planning and Quick Intervention.** Effective planning greatly contributed to the success of safety and health protections during the response. In March 2010, the month before the oil spill, OSHA and other agencies participated in the Spill of National Significance 2010 exercise. This exercise helped forge effective coordination among members of the NRT. In particular, the exercise helped OSHA and USCG define their respective roles and responsibilities during an oil spill response. When the spill occurred in April, the experience from the exercise resulted in OSHA’s early activation and easier integration into the operational command structure.

For several years prior to the Deepwater Horizon response, NIOSH led an interagency workgroup to develop national guidance on protecting workers before, during, and after a response. The workgroup was a consortium of federal agencies, including OSHA and the USCG, state health departments, and volunteer organizations. The group produced the Emergency Responder Health Monitoring and Surveillance (ERHMS) draft guidance document, which was instrumental in building collaborations and preparing agencies for a response of this magnitude.^16^

Even before the disaster, some members of the NRT had plans and procedures to address some of the hazards that were present during the oil spill response. These protocols were useful as resources for tailoring protections to this response. For example, USCG had a heat stress program, and their experience and expertise in this area was invaluable when making recommendations to BP for creating its heat stress program.^11^ After the spill occurred, members of the NRT developed guidance specific to the response, including the OSHA/NIOSH guidance document addressing chemical hazards and the use of respiratory protection.^8^ To the extent that hazards can be anticipated, having prototype procedures and guidance would help expedite the implementation of safety and health plans and worker protections.

In the Deepwater Horizon response, it took weeks for the oil to travel from the source to the shoreline, which gave responders enough time to establish staging areas and to implement controls and safe work practices well before the oil reached the shore. Response agencies will rarely have weeks to prepare for an emergency. However, it is clear that advance planning and rapid deployment greatly contribute to the success of worker safety and health protections during a response.

**Collaborative Efforts.** OSHA and NIOSH’s efforts to protect workers would have not have been as successful without close collaborations with other agencies. OSHA worked closely with USCG, BP, and local, state, and federal health agencies to jointly plan and execute response actions and worker safety and health programs. These collaborations were highly beneficial in terms of gaining situational awareness, coordinating response efforts, and ensuring that safety and health issues were identified and effectively addressed.

Collaborations helped resolve deficiencies that OSHA observed in BP’s safety and health program at several work sites and staging areas throughout the Gulf Coast region. OSHA staff observed inconsistent implementation of safety measures, including: gaps in BP’s heat stress program; a lack of plans for addressing inclement weather, workplace violence, and site control; and delays in abating worker hazards and in sharing worker injury and exposure data. Working through the Unified Command, OSHA brought these issues and concerns to the attention of the USCG Federal On-Scene Coordinator and the NIC Commander, provided timely feedback to BP on its incident-wide site safety and health plan, and made sure that BP incorporated supplemental plans addressing specific worker hazards and corrected observed hazards. The USCG and OSHA signed a Memorandum of Understanding to institutionalize this cooperative arrangement and demonstrate the command’s commitment to worker safety and health.

The agencies involved brought different expertise and resources to worker protection efforts. NIOSH staff, including its Epidemic Intelligence Service officers, managed the rostering effort, analyzed illness and injury data and conducted Health Hazard Evaluations of specific occupational risks, including investigation of reports of symptoms or exposure effects from exposed workers.^9^ ^17^ OSHA and NIOSH published guidance on recommended personal protective equipment, exposure assessments, heat stress, and medical care. OSHA and NIEHS provided oversight of the training that BP and its contractors conducted. This collaborative effort extended to the development of training publications, including the widely distributed Safety and Health Awareness for Oil Spill Cleanup Workers pocket booklet,^12^ as well as the review of training materials.

OSHA assisted the Unified Command in assessing potential chemical exposures during off-shore operations. In addition, OSHA provided assistance to USCG’s Industrial Hygiene group, including providing sample media, equipment, sampling and analytical methods, and laboratory analysis of USCG’s samples (including posting of the results on OSHA’s website). OSHA partnered with NOAA for additional sampling for potential chemical exposures during NOAA’s off-shore research.

**Alternatives to OSHA Enforcement.** The response to the Deepwater Horizon oil spill also highlighted the benefits of using what OSHA terms “technical assistance,” rather than enforcement as the primary approach during a response in which all parties are committed to full implementation of worker protection protocols and measures. Under normal situations, OSHA’s compliance officers enforce the agency’s Permissible Exposure Limit (PEL) standards, many of which are out of date, but have the force of law. In the Deepwater Horizon response, instead of solely enforcing the OSHA PELs, OSHA used its technical assistance role to encourage the Unified Command to base protection decisions on the most protective occupational exposure limits available, such as the Recommended Exposure Limits (RELs) developed by NIOSH, and the Threshold Limit Values (TLVs) recommended by the ACGIH. OSHA also worked with BP and USCG to implement a best-practice, comprehensive, incident-wide heat stress plan, even though OSHA does not have a specific standard addressing the hazard of heat. In contrast, OSHA’s enforcement role would have only provided protections based on the existing PELs, and could have led to litigation and potentially delayed the abatement of hazards.

While the agency retained the option of moving into an enforcement role, OSHA operated in a technical assistance role during the Deepwater Horizon response and believes that was the most effective role for this particular response. During the oil spill response, under the Unified Command, BP was required to immediately address worker safety and health issues. OSHA believes this contributed to the low rate of injury among cleanup workers.

**Openness and Transparency.** Environmental emergencies like the Deepwater Horizon oil spill can create a potential for great misunderstanding. In the confusion and uncertainty after a disaster, fear and mistrust can be exacerbated if there is incomplete data disclosure or if the data are not presented in an easily understood format. In this incident, stakeholders’ initial lack of access to exposure monitoring data led to misconceptions regarding worker protections.

One example of how poorly presented information can affect the perception of a response involved BP sampling data. A national newspaper published an article highlighting a BP report that stated that 80 percent of their samples showed “no detectable level” of the dispersant component 2-butoxyethanol, and that the other 20 percent of the samples were detected at a level below 10 ml/m3.^18^ The article’s headline read alarmingly “New BP Data Show 20% of Gulf Spill Responders Exposed to Chemical That Sickened Valdez Workers”. OSHA reviewed the data and found that of that 20 percent, the highest level recorded was 0.8 ml/m3 and that 90 percent of the samples were at or below 0.2 ml/m3. These levels were well below the lowest OEL for 2-butoxyethanol, NIOSH’s REL of 5 ppm. OSHA’s independent samples were all below the limit of detection for 2-butoxyethanol. Despite the fact that all of the samples were well below the lowest OEL, BP’s choice of words to report the data led to a misleading interpretation of the data.

The Deepwater Horizon response also demonstrated the importance of presenting data with a description of the precautions being used in the area where a sample was taken. For example, 89 percent of the 2-butoxyethanol results above the detectable level were taken near the source of the oil spill where dispersants were being sprayed. Controls, including respiratory protection, were implemented to protect workers near the source. Because protections were in place, these exposure measurements did not reflect doses absorbed by workers.

Although the context could have been more clearly communicated, OSHA made a large amount of exposure data available to the public and insisted that BP post its sampling results on the Internet. Encouraging the parties involved in a response to make their data readily accessible is a simple way to promote transparency and foster trust with the affected community.

**Communication.** Although the agencies successfully reached out to members of the impacted communities, they faced challenges getting their message out to the public as a whole. This is a classic public health communications problem – successful public health work has no victims, while media stories tend to focus on victims, mistakes and scandals. Even though worker safety and health efforts, such as the heat stress mitigation program, likely prevented injuries and deaths, there is no way to identify or quantify the workers whose lives were saved. There was significant media attention on initial reports regarding seven fishermen who reported symptoms that potentially could have been related to chemical exposures. However, there was little attention paid to NIOSH’s investigation, which found that it was unlikely that exposure to dispersants caused the fishermen’s symptoms. The media also did not focus on the lack of symptoms among the thousands of cleanup workers employed for the duration of the response; as a result, many Americans were left with the false impression that chemical-related symptoms were common among workers employed in the cleanup.

## Conclusion

Several factors contributed to the successful efforts to protect workers during the Deepwater Horizon oil spill response. Advance planning and immediate deployment, collaboration across agencies, more protective safety and health protections than required by regulation, and community outreach helped ensure that BP implemented controls to protect workers and properly trained workers to perform their jobs safely. For future responses, improvements should be made in how safety and health information, and the bases for safety and health decisions, are communicated to the public. OSHA and NIOSH’s efforts alongside other agencies helped ensure that the response and cleanup operations resulted in no worker fatalities, and relatively few injuries and illnesses.

## References

[1] United States Coast Guard National Incident Command. National Incident Commander’s Report: MC252 Deepwater Horizon. 2010.

[2] National Commission on the BP Deepwater Horizon Oil Spill and Offshore Drilling. Decision-Making Within the Unified Command. 2010.

[3] Occupational Safety and Health Act of 1970. 1970. Public Law 91-596.

[4] National Oil and Hazardous Substances Pollution Contingency Plan. 1994. 40 C.F.R. 300.

[5] OSHA (Occupational Safety and Health Administration). Emergency Response to Oil Spill Initial Sampling Strategy. 2010.

[6] OSHA (Occupational Safety and Health Administration). Assessing Worker Exposures. 2010.

[7] BP p.l.c. Health monitoring. 2010.

[8] NIOSH (National Institute for Occupational Safety and Health) and OSHA (Occupational Safety and Health Administration). Interim Guidance for Protecting Deepwater Horizon Response Workers and Volunteers. 2010.

[9] NIOSH (National Institute for Occupational Safety and Health). Deepwater Horizon Response NIOSH Health Hazard Evaluation. 2010.

[10] Deepwater Horizon MC252 New Orleans, LA (NOLA) Unified Area Command (UAC). 208 Heat Stress Safety Alert. 2010.

[11] Deepwater Horizon MC252 New Orleans, LA (NOLA) Unified Area Command (UAC). Heat Stress Management Plan. 2010.

[12] OSHA (Occupational Safety and Health Administration) and NIEHS (Nationial Institute for Environmental Health Sciences). Safety and Health Awareness for Oil Spill Cleanup. 2010.

[13] Grand Isle Sees First Oil As Tar Balls Wash Ashore: Heavy Crude Seen In Elmer's Island.

[14] Kitt MM, Decker JA, Delaney L, et al. Protecting workers in large-scale emergency responses: NIOSH experience in the deepwater horizon response. J Occup Environ Med. 2011;53(7):711-715. 10.1097/JOM.0b013e31822543b6 21697736

[15] Gulf Long-Term Follow-Up Study for Oil Spill Clean-Up Workers and Volunteers.

[16] NIOSH (National Institute for Occupational Safety and Health). Emergency Responder Health Monitoring and Surveillance: Draft 1.2. 2010.

[17] NIOSH (National Institute for Occupational Safety and Health). NIOSH Report of Deepwater Horizon Response/Unified Area Command Illness and Injury Data (April 23 – July 27, 2010).

[18] Schor, Elana. New BP Data Show 20% of Gulf Spill Responders Exposed to Chemical That Sickened Valdez Workers. New York Times. July 9, 2010.

